# Contribution of colony-stimulating factor 1 to neuropathic pain

**DOI:** 10.1097/PR9.0000000000000883

**Published:** 2021-03-09

**Authors:** Xiaobing Yu, Allan Basbaum, Zhonghui Guan

**Affiliations:** Deaprtments of a Anesthesia and Perioperative Care and; b Anatomy, University of California San Francisco, San Francisco, CA, United States

**Keywords:** Neuropathic pain, microglia, macrophage, spinal cord, dorsal root ganglion

## Abstract

Molecular and cellular interactions among spinal dorsal horn neurons and microglia, the resident macrophages of the central nervous system, contribute to the induction and maintenance of neuropathic pain after peripheral nerve injury. Emerging evidence also demonstrates that reciprocal interactions between macrophages and nociceptive sensory neurons in the dorsal root ganglion contribute to the initiation and persistence of nerve injury-induced mechanical hypersensitivity (allodynia). We previously reported that sensory neuron-derived colony-stimulating factor 1 (CSF1), by engaging the CSF1 receptor (CSF1R) that is expressed by both microglia and macrophages, triggers the nerve injury-induced expansion of both resident microglia in the spinal cord and macrophages in the dorsal root ganglion and induces their respective contributions to the neuropathic pain phenotype. Here, we review recent research and discuss unanswered questions regarding CSF1/CSF1R-mediated microglial and macrophage signaling in the generation of neuropathic pain.

## Abstract 2

This review discusses recent research and the unanswered questions regarding CSF1/CSF1R-mediated microglial and macrophage signaling in the generation of neuropathic pain.

## 1. Introduction

Persistent pain, including neuropathic pain, has a profound social and economic impact on society.^26^ A particularly active area of research into the mechanisms that contribute to the transition from acute to chronic pain after peripheral nerve injury focuses on the interactions between neurons and immune cells in both the central and peripheral nervous system.^2,52^ Microglia and macrophages, which are, respectively, the principal immune cells in the central nervous system (CNS)^27^ and peripheral nervous system,^83^ rapidly expand after peripheral nerve injury. Furthermore, many recent studies demonstrated that complex molecular and cellular interactions between microglia and spinal dorsal horn neurons contribute to the development of neuropathic pain after peripheral nerve injury.^25,27^ Cross-talk between macrophages and nociceptive sensory neurons in the dorsal root ganglion (DRG) has similarly been implicated in the initiation and persistence of nerve injury-induced mechanical hypersensitivity, a hallmark of the neuropathic pain phenotype.^5,41^ With an understandable interest in identifying the cellular basis of injury-induced expansion of immune cells, we recently reported that sensory neuron-derived expression of colony-stimulating factor 1 (CSF1) after nerve injury triggers the activation of both resident microglia in the spinal cord^16^ and resident macrophages in the DRG^80^ through binding the CSF1 receptor (CSF1R) that is expressed by these cells. Here, we review recent research advances and discuss several unanswered questions regarding CSF1/CSF1R signaling and the regulation of microglia and DRG macrophages. Of particular interest are male–female differences that underlie a profound dimorphism in the immune cell contribution to nerve injury-induced neuropathic pain.

## 2. CSF1 and its receptor, CSF1R

CSF1, also known as macrophage colony-stimulating factor (M-CSF), was the first isolated growth factor that stimulates the differentiation of bone marrow hematopoietic stem cells/progenitors into the macrophage lineage.^68^ Other CSFs were subsequently discovered. These include CSF2, also known as granulocyte-macrophage colony-stimulating factor (GM-CSF), which stimulates granulocyte and macrophage colony formation, and CSF3, also known as granulocyte colony-stimulating factor (G-CSF), which stimulates granulocyte colony formation.^43^ Further studies demonstrated that CSF1 also activates cultured primary microglia.^60^ The translational relevance of these findings was significantly increased after the discovery of a natural mutation, in osteopetrotic (*op*) mice, of the gene that encodes CSF1 (*Csf1*).^79^ Microglia density is significantly reduced in multiple brain regions of *op/op* mice,^11,33^ which suggests that CSF1 is an essential contributor to the development and/or the maintenance of microglia in the CNS.

The receptor targeted by CSF1, CSF1R, is a member of the type III protein tyrosine kinase receptor family.^24^ An early immunohistochemistry (IHC) study reported that CSF1R protein is expressed in microglia in the brain and spinal cord,^55^ and a later study in *Csf1r*-GFP reporter mice found that the *Csf1r* gene, in the brain, is expressed in microglia, but not in neurons, astrocytes, or oligodendrocytes.^10^ Subsequent study found that the *Csf1r* gene is expressed in microglia at an early embryonic stage in the yolk sac and that expression of the *Csf1r* gene is sustained throughout microglia development.^11^ The CSF1R is also critical for microglia development; microglia are almost completely absent in *Csf1r* mutant mice.^10,11^ Moreover, maintenance of adult microglia requires CSF1R signaling; pharmacological inhibition of CSF1R in the adult largely eliminates microglia.^9^

Somewhat surprisingly, perhaps, the microglia deficit in *Csf1r* mutant mice is far more severe than what occurs in *Csf1* mutant *op/op* mice.^11^ This finding suggests that CSF1 is not the sole ligand for microglial CSF1R. In fact, interleukin-34 (IL-34) is a second cognate ligand of CSF1R.^39^ Interestingly, the IL-34 gene, *Il34,* is expressed at much higher level and in broader regions of adult brains than *Csf1*.^45,74^ As for the *Csf1* mutant *op/op* mice, *Il34* mutant mice also have a lower microglia density in the adult, but again, not as profound as the deficit that occurs in *Csf1r* mutant mice.^14,73^ Unlike CSF1, whose only target is the CSF1R, IL-34 can also bind receptor-type protein-tyrosine phosphatase ζ, a cell surface chondroitin sulfate proteoglycan that is expressed in multiple brain regions.^44^

## 3. De novo induction of CSF1 in sensory neurons

Spinal microglia activation has long been considered a significant contributor to neuropathic pain after peripheral nerve injury.^4^ Insight into mechanisms that lead to the neuropathic pain phenotype, however, came from the appreciation that nerve damage does not always lead to spinal cord microglia activation. Thus, although injury to the peripheral axonal branch of primary sensory neurons reliably produces spinal dorsal horn microglia activation, denervation produced by dorsal rhizotomy, namely transecting or ligating the central branch of the peripheral nerve (dorsal root), between the DRG and spinal cord, produces much less dorsal horn microglia activation.^7,40^ This critical distinction led to the suggestion that injured sensory neurons must send signals to the dorsal horn of the spinal cord to activate microglia.

Searching for this signal, we performed RNA sequencing (RNA-Seq) of ipsilateral and contralateral DRG and lumbar dorsal horn from naive mice and from mice 7 days after sciatic nerve transection. Our experiments were designed to identify potential communication signals between the DRG and spinal dorsal horn, signals that might be the trigger for microglial activation.^16^ We found that the *Csf1* gene was among the most highly enriched genes in the DRG ipsilateral to the peripheral nerve injury and that the gene encoding the CSF1 receptor, *Csf1r*, was highly enriched in the ipsilateral dorsal horn after the injury. Quantitative RT-PCR (qRT-PCR) confirmed the *Csf1* induction in the ipsilateral DRG and demonstrated that the induction occurs within 1 day after nerve injury, and it persists for at least 3 weeks after the injury. Interestingly, although *Il34* is expressed in the DRG, its expression did not change after nerve injury. Finally, by in situ hybridization and IHC, we demonstrated that both *Csf1* mRNA and CSF1 protein are induced in ATF3-positive, ie, axotomized DRG neurons of all diameters, and that all the sensory neurons immunoreactive for CSF1 protein coexpress ATF3. On the other hand, we found that ∼80% of the ATF3-positive DRG neurons are CSF1^(+)^ 1 day after nerve injury, and the percentage of CSF1 coexpressed ATF3-positive DRG neurons drops to ∼40% 3 weeks after nerve injury. Whether it persists for longer periods is not clear. Most importantly, to determine whether newly synthesized, sensory neuron-derived CSF1 protein is transported to the lumbar dorsal horn, we ligated the L4 and L5 dorsal roots at the time of sciatic nerve transection. Although this is a difficult experiment, with immunohistochemistry, we found that CSF1 protein accumulates at the proximal end of the ligature, which suggests that CSF1 protein is indeed transported through the dorsal root to the spinal cord.

In parallel studies, the Noguchi laboratory investigated expression of the CSF family in the DRG.^48^ These authors found that *Csf1* and *Il34*, but neither *Csf2* nor *Csf3*, are expressed in rat DRG. Similar to our findings, they found that *Csf1* is induced in the DRG 1 day after spared nerve injury (SNI) and persists for at least to 14 days, whereas the expression of *Il34* does not change. By in situ hybridization, they demonstrated that *Csf1* mRNA is expressed in few DRG neurons in naive rat, but in ∼40% of small, ∼43% of medium, and ∼60% of large DRG neurons 2 days after SNI. By comparison, they found that ∼58% of all DRG neurons are ATF3 (+) at the same time point after the nerve injury. By combined in situ hybridization and immunostaining, they found that ∼96% of *Csf1* mRNA (+) DRG neurons coexpress ATF3, whereas ∼63% of ATF3 (+) DRG neurons coexpress *Csf1* mRNA 2 days after the injury. The in situ hybridization study also showed that *Il34* mRNA is expressed in non-neuronal cells that surround the DRG neurons and that its expression is not influenced by the injury.

Recent studies provided further evidence for the induction of the *Csf1* gene and CSF1 protein in injured sensory neurons, in both the mouse and rat SNI models. Importantly, these studies recorded upregulation of *Csf1* gene that persisted for at least 6 weeks after the injury.^47,75^ Of note, *Csf1* gene induction is not limited to the SNI neuropathic pain model. For example, *Csf1* gene is upregulated in the rat DRG 7 to 14 days after chronic constriction injury (CCI),^47,69^ and CSF1 protein is induced in ∼55% ATF3^(+)^ DRG sensory neurons 7 days after the surgery.^47^ The *Csf1* gene is also upregulated in trigeminal ganglion 21 days after infraorbital nerve CCI.^34^ Interestingly, although the *Csf1* gene is induced in DRG after CCI in both male and female animals, the *Csf1* induction in female animal is greater.^34,69^ In a mouse model of lumbar disc degeneration, CSF1 protein is induced in L3 CGRP-expressing DRG neurons 2 weeks after L3/L4 disc puncture.^77^ Moreover, facial tissue injury, without apparent injury to the facial nerve, can induce *Csf1* gene expression in ATF3 (+) trigeminal ganglion sensory neurons.^46^ Finally, a single-cell sequencing study of DRG neurons recently demonstrated that *Csf1* induction in injured DRG neurons is reduced when the *Atf3* gene is deleted from sensory neurons,^57^ suggesting that ATF3 is at least partially responsible for *Csf1* induction after nerve injury.

On the other hand, there are reports that CSF1 can be induced in sensory neurons without concurrent ATF3 induction. That finding suggests that frank nerve damage may not be required.^82^ For example, high-frequency stimulation (100 Hz, 10 V) of the mouse sciatic nerve, which induces long-term potentiation in the dorsal horn of the spinal cord and long-lasting mechanical hypersensitivity, induces *Csf1* gene and CSF1 protein in sensory neurons without obvious nerve damage, although at much lower levels than what occurs in the SNI model.^82^ Even stimulation of cultured DRG neurons with 40 mM KCl for 24 hours can increase CSF1 protein production in the culture medium.^82^

## 4. Contribution of CSF1 to neuropathic pain

To investigate the function of neuronally induced CSF1 to neuropathic pain, we deleted *Csf1* genes from DRG sensory neurons by crossing *Csf1*^*fl/fl*^ mice^18^ with *Adv-Cre* mice^84^ and monitored nerve injury-induced behavior. We found that mice in which *Csf1* was deleted from sensory neurons do not develop mechanical hypersensitivity in the SNI model. We concluded, therefore, that sensory neuron-derived CSF1 is required for the development of neuropathic pain behavior.^16^ Importantly, deleting *Csf1* from sensory neurons is remarkably selective in its influence on the mice. We found no changes in baseline mechanical sensitivity, formalin-induced inflammatory pain behavior, response to noxious heat stimulation, motor activity, or body weight of the animal.^16^ Focusing on the downstream consequences of CSF1 induction, we next examined the effects of intrathecal injection of CSF1. By itself, intrathecal CSF1 protein provokes a significant mechanical hypersensitivity in mice,^16^ a finding independently confirmed by other groups, in both the mouse and rat.^48,78,82^ Based on these results, we conclude that intrathecal CSF1 is sufficient to induce pain behavior. Consistent with this conclusion, although *Adv-Cre; Csf1*^*fl*/fl^ mutant mice fail to develop mechanical hypersensitivity after nerve injury, in these mice, intrathecal CSF1 protein nevertheless provoked significant mechanical hypersensitivity.^16^ This finding indicates that CSF1 is sufficient to induce the neuropathic pain phenotype.

Interestingly, in a pain model in which mechanical hypersensitivity is induced by 10 V, 100-Hz high-frequency stimulation of the mouse sciatic nerve, without causing obvious injury to DRG sensory neurons, intrathecal injection of CSF1 antibody blocks the development of mechanical hypersensitivity.^82^ Local application of CSF1 antibody onto the spinal cord section also blocks the high-frequency stimulation-induced long-term potentiation in the spinal cord dorsal horn.^82^ Based on these diverse findings, we conclude that induction of CSF1 in sensory neurons is a necessary contributor to the neuropathic pain phenotype after peripheral nerve injury, even in conditions in which frank nerve damage is avoided.

## 5. CSF1 activates spinal cord microglia and induces pain behavior through CSF1R expressed in microglia

As CSF1 protein is transported from the DRG to the spinal cord,^16^ it is clearly of interest to determine the cell type acted upon by CSF1 in the spinal cord. A variety of studies are consistent with previous reports that the CSF1 receptor, CSF1R, is expressed only in microglia, not in neurons, astrocytes, or oligodendrocytes in brain.^10^ The confirmation came from studies in *Csf1r*-GFP reporter mice, in situ hybridization, and IHC, which all demonstrated that the *Csf1r* gene and CSF1R protein are expressed in spinal cord microglia of both mouse and rat, and that it is upregulated in the lumbar cord after various nerve injuries, puncture of lumbar disc, or high-frequency stimulation of the sciatic nerve injury.^16,48,77,82^ Moreover, deletion of *Csf1* from sensory neurons prevents nerve injury-induced microglia activation,^16^ suggesting that sensory neuron-derived CSF1 is required for microglia activation after nerve injury. Finally, intrathecal injection of CSF1 protein activates microglia and induces several key microglia marker genes that are upregulated after peripheral nerve injury,^25^ including *Itgam* (encoding CD11b), *Cx3cr1*, *Bdnf*, and *Ctss* (encoding CatS).^16^ The latter result is significant because it indicates that CSF1, by itself, is not only sufficient to trigger mechanical hypersensitivity but also to activate spinal cord microglia.

The significance of the CSF1-CSF1R connection to the nerve injury-induced neuropathic pain phenotype is most strongly supported by antagonist studies. For example, intrathecal CSF1 injection-induced mechanical hypersensitivity can be blocked by minocycline,^16^ a nonselective microglial inhibitor. Furthermore, inhibition of CSF1R with GW2580, a selective CSF1R antagonist, administered either before or immediately after nerve injury, significantly suppressed the early phase of SNI-induced mechanical hypersensitivity in the rat. On the other hand, when it was administered 12 days after SNI,^48^ GW2580 was without effect on the late phase of the mechanical hypersensitivity, even though neuronal CSF1 expression in the DRG remains elevated at this time.^47^ Taken together, these studies suggest that signaling through CSF1R is an important contributor to the initiation, but not to the maintenance of the neuropathic pain phenotype after nerve injury. This conclusion is also consistent with the fact that microglia activation is most prominent in the early phase after peripheral nerve injury,^28^ and that the effect of minocycline is also limited to the early phase of the nerve injury-induced mechanical hypersensitivity.^54^

## 6. A microglial DAP12/TREM2 pathway contributes to neuropathic pain

Unclear, of course, is the mechanism through activation of microglia influences dorsal horn pain transmission circuits. In fact, even the signaling pathways downstream of microglial CSF1R that are engaged in the setting of nerve injury are not fully understood. Our own studies provided evidence for a necessary contribution of DNAX-activating protein of 12 kDa (DAP12, even though a type I transmembrane polypeptide that contains an immunoreceptor tyrosine-based activation motif.^36^ DAP12, which plays a central role in microglia signaling transduction,^20^ is engaged downstream of CSF1R in bone marrow-derived macrophages.^49^ Given its relevance to microglial signal transduction, we hypothesized that DAP12 also participates in CSF1-induced and nerve injury-induced pain. Several studies, in fact, support this hypothesis. For example, *Tyrobp* gene, which encodes DAP12 protein, is induced in the lumbar spinal cord after intrathecal CSF1 injection,^16^ in dorsal horn microglia after L4 spinal nerve injury,^31^ as well as in microglia in the hypoglossal nucleus after XIIth nerve injury.^32^ DAP12 protein is also upregulated and phosphorylated in lumbar spinal cord microglia after peripheral nerve injury.^31^ Most importantly, we and others found that nerve injury and intrathecal CSF1-induced microglia gene upregulation and neuropathic pain behavior are prevented or reduced in DAP12 knockout mice,^16,31^ which suggests that DAP12 lies downstream of CSF1R in the microglia that contribute to nerve injury-induced neuropathic pain.

Other studies suggest that DAP12 is engaged in association with TREM2, the triggering receptor expressed on myeloid cells 2 protein, a well-established modulator of microglia function.^42,71^ As for DAP12, *Trem2* gene is induced in dorsal horn microglia after L4 spinal nerve injury,^31^ and activation of TREM2 by intrathecal injection of a TREM2 agonist antibody activates spinal cord microglia and induces mechanical hypersensitivity.^31^ Interestingly, the TREM2 activation-induced microglial gene upregulation and pain behavior is DAP12-dependent.^31^ In addition, inhibition of TREM2 with a neutralizing antibody significantly attenuates chemotherapy-induced neuropathic pain behavior.^21^

## 7. Contribution of CSF1-CSF1R to maintenance and self-renewal of spinal microglia

Microglia originate from yolk sac and migrate into developing brain and spinal cord early in embryonic development.^11,62^ In the adult, microglia maintain their population by a self-renewal process, without a contribution of peripheral monocytic cells.^64^ Consistent with that process, recent studies concluded that circulating monocytes do not contribute to the expansion of lumbar cord microglia after peripheral nerve injury. Of particular interest, our qRT-PCR study demonstrated that although microglia specific genes are upregulated, peripheral monocyte specific genes could not be detected in the lumbar cord after sciatic nerve injury.^16^ Similarly, studies in a CCR2 reporter mouse, which marks a gene that is exclusively expressed in peripheral monocytes,^59^ found no evidence for infiltration of peripheral monocytes into the spinal cord after nerve injury.^15,17,31^ And most interestingly, parabiosis studies, in which 2 mice are surgically joined to share circulating blood cells,^76^ confirmed that there is no peripheral monocyte infiltration to the spinal cord after nerve injury.^17,70^ Taken together, these results overwhelmingly demonstrate that proliferative self-renewal of microglia, rather than monocyte infiltration, underlies the microglia expansion that occurs in the spinal cord after peripheral nerve injury.

Not surprisingly, perhaps, we also established that sensory neuron-derived CSF1 is critical to the spinal cord microglia proliferation after nerve injury. Deleting *Csf1* from sensory neurons, or intrathecal injection of the CSF1R inhibitor, GW2580, greatly reduces nerve injury-induced microglia proliferation in the spinal cord,^16,48^ while intrathecal injection of CSF1 induces spinal microglia proliferation.^16,48^ Importantly, however, the effects of these manipulations were never complete; a low level of nerve injury-induced microglia proliferation in the spinal cord persists in the mice with *Csf1* deleted from sensory neurons or after intrathecal CSF1R inhibition.^16,48^ Those results suggest that sensory neuron-derived CSF1 is not the only signal that stimulates microglia proliferation after nerve injury.

CSF1 is also crucial for maintaining a homeostatic microglial population, namely baseline levels. Although deletion of *Csf1* from sensory neurons has little effect on the baseline microglia population in the spinal cord, deletion of *Csf1* from CNS neurons with *Nestin-Cre* substantially reduces microglia baseline density in the spinal cord,^16^ suggesting that neuronal *Csf1* in CNS is important in maintenance of microglial homeostatic status. Furthermore, treating an animal with the CSF1R inhibitor, PLX3397 or PLX5622, which are more potent than GW2580, profoundly depletes CNS microglia,^9,67^ and the recovery from these manipulations results in a dramatic microglia proliferative repopulation.^23,81^ The exact mechanism downstream of CSF1R for microglia proliferation remains to be elucidated.

## 8. Induction of CSF1 in injured motor neurons

Peripheral nerve injury not only transects peripheral axons that derive from DRG sensory neurons but also from spinal cord motoneurons. As for the sensory neurons, CSF1 is induced in ATF3 (+) motor neurons in the ventral horn of the lumbar spinal cord after sciatic nerve injury. Moreover, the de novo synthesized CSF1 distributes from the cell bodies to motoneuron dendrites and axons, extending peripherally and accumulating at the site of nerve injury.^16^ Importantly, motoneuron-derived CSF1 is also an important contributor to nerve injury-induced activation and proliferation of microglia in the vicinity of the axotomized motoneurons. In fact, we found that this microglia proliferation is greatly reduced in mice with *Csf1* deleted from all CNS neurons.^16^ In addition, VIIth nerve injury-induced microglia activation and proliferation in the facial nucleus is also greatly reduced in *Csf1* mutant *op/op* mice.^30^

## 9. Contribution of dorsal root ganglion macrophages to neuropathic pain

Paralleling the activation of dorsal horn microglia after peripheral nerve injury is a concurrent and persistent expansion of macrophages in the DRG ipsilateral to the injury.^22,61,65,80^ To what extent the increase of DRG macrophages contributes to the initiation and the maintenance of nerve injury-induced mechanical hypersensitivity remains a major area of research. To a great extent, the significant discrepancies in the literature result from the technical challenge of conditional killing of macrophages, without impacting microglial survival. For example, selective CSF1R antagonists readily cross the blood–brain barrier (BBB) and thus target both CNS microglia^9,37,53^ and peripheral macrophages.^37^ The same limitation also applies to several transgenic mouse lines that express a drug-inducible suicide gene, eg, herpes simplex virus type 1 thymidine kinase (CD11b-TK)^19^ or diphtheria toxin receptor (CD11b-DTR,^8^ LysM-DTR,^13^ and Cx3cr1-DTR^50^), in both microglia and macrophages. A different approach used clodronate, which can target both macrophages and microglia cells, but because of its size, it has a more limited capacity to cross the BBB to kill microglia. To date, however, there is little agreement as to the postdepletion behavioral phenotype after nerve injury.^1,6,38,51,58^ We presume that the lack of agreement reflects variability in the efficacy of clodronate.

An alternative approach involves use of the Macrophage Fas-Induced Apoptosis (MAFIA) transgenic mouse line^3^ that expresses a suicide gene, *Fas*, under the control of the CSF1R promoter. Selective depletion of peripheral macrophages can be achieved with an FK-binding protein dimerizer, AP20187 (AP), which does not cross the BBB.^3,80^ Shepherd et al.^63^ used MAFIA mice to deplete circulating monocytes and macrophages and reported reduced nerve injury-induced mechanical hypersensitivity. The authors found that macrophages in the DRG were intact and therefore concluded that peripheral macrophages, not those in the DRG, are the critical contributors to neuropathic pain.^63^ Their findings, however, disagreed with those of an earlier study that used a different approach to selectively kill peripheral circulating monocytic cells, while sparing DRG macrophages, and found a limited impact on neuropathic pain development.^51^

Given the little consensus as to whether the macrophage population in the DRG or at the nerve injury site is more relevant to nerve injury-induced pain initiation, we also investigated the utility of the MAFIA to re-examine the question.^80^ In these studies, we administered AP systemically, before or after producing the peripheral nerve injury. This approach made it possible to examine the contribution of peripheral macrophages to both the initiation and the maintenance of the nerve injury-induced mechanical hypersensitivity. Most importantly, we found that both resident macrophages in the DRG and peripheral monocytic cells can be significantly depleted by AP treatment in the MAFIA mice and that the mechanical hypersensitivity could be prevented and in fact, reversed when the AP was administered after the hypersensitivity had developed. Furthermore, we confirmed that the approach spared spinal cord microglia. To distinguish the contribution of DRG macrophages from those migrating to the site of the peripheral nerve injury, in a separate set of studies, we selectively depleted macrophages at the injury site, by local AP application. In these studies, we found that depletion of macrophages in the DRG, but not at the peripheral nerve injury site, prevents the development of and reverses persistent nerve injury-induced mechanical hypersensitivity, in both male and female mice. Our findings are of interest in light of the report of Sorge et al. that microglial depletion in male, but not female mice, reduced nerve injury-induced neuropathic pain.^66^ Given that the contribution of the DRG macrophages to the mechanical hypersensitivity seems not to be sexually dimorphic, our findings suggest that the cellular interaction between macrophages and sensory neurons in the DRG are a potential target for future neuropathic pain management in both males and females.

A question still remains whether peripheral injury-induced macrophage expansion in the DRG results from infiltration of circulating monocytic cells or depends on resident macrophage proliferation. Previously, ED1^+^ macrophages identified in rat cervical ganglia were believed to be infiltrating cells.^61^ A comparable conclusion derived from studies demonstrating expression of another purported marker of infiltrating macrophages, namely CCR2. On the other hand, CCR2^+^ macrophages are also found in the DRG of uninjured mice.^72,80^ Furthermore, in a recent report, Wang et al.^72^ studied a parabiosis model in naive mice and concluded that in the absence of nerve injury, there was minimal contribution of circulating cells from the parabiotic partner. The authors also conducted pulse-chase labeling of peripheral macrophages in *Csf1r*^Mer-iCre-Mer^ x *tdTomato*^fl/fl^ mice, in which reporter gene expression in *Csf1r*^*+*^ cells could be triggered with tamoxifen to induce Cre recombination. As expected, both peripheral monocytic cells and microglia in the CNS, which were CSF1R^+^, became tdTomato^+^. Eight weeks after tamoxifen removal, more than 80% of the circulating monocytes were gradually replaced by BM-derived hematopoietic progenitors and lost reporter gene expression (tdTomato^+^). By contrast, 96% of peripheral macrophages in the DRG remained positive for tdTomato, suggesting there is minimal contribution from circulating monocytic cells, which were already negative for tdTomato. Rather, those DRG resident macrophages were self-maintained or derived from resident cells that proliferate in the naive animal.

In the context of nerve injury, however, there may be a compromised blood–nerve barrier in the DRG after nerve injury that could result in increased permeability^56^ and potentially allow the recruitment of circulating monocytic cells to contribute to the expansion of DRG macrophages. Because of the controversy, we initiated experiments directed at origin of the injury-induced macrophage expansion in the DRG. In these studies, we used Ki67 immunostaining to monitor proliferating CX3CR1^+^ macrophages after nerve injury.^80^ We found that at 24 hours after nerve injury, there was no difference in the percentage of Ki67^+^CX3CR1^+^ macrophages in the ipsilateral, denervated DRG from the uninjured contralateral DRG. However, 4 days after the nerve injury, the percentage of Ki67^+^CX3CR1^+^ macrophages more than doubled in the ipsilateral DRG. We cannot completely rule out a contribution of infiltrating macrophages because these cells may retain their Ki67 phenotype. However, based on our finding that the percentage of Ki67^+^CX3CR1^+^ macrophages did not change within the first 24 hours after the injury, we conclude that injury-induced macrophage expansion in the DRG predominantly involves local proliferation of resident macrophages.

## 10. CSF1 redefines the collaborative contribution of spinal microglia and dorsal root ganglion macrophages to neuropathic pain

Of particular interest is a possible interaction of the de novo expression of CSF1 in axotomized DRG neurons and the proliferating macrophages. As DRG macrophages also express CSF1R, we re-examined *Adv-Cre; Csf1*^*fl*/fl^ mice, in which *Csf1* gene expression is depleted selectively from sensory neurons.^16^ As described above, these mice do not develop the nerve injury-induced neuropathic pain phenotype. In these mice, we also found that conditional deletion of *Csf1* abolished the injury-induced expansion of macrophages in the ipsilateral DRG.^80^ Most interestingly, we found that there is a sexual dimorphism in the contribution of CSF1 to the nerve injury-induced expansion of DRG macrophages. Specifically, deletion of CSF1 in sensory neurons only affected macrophage expansion in the DRG of male mice. It remains to be determined whether other nerve injury-induced factor released in the axotomized sensory neurons influence macrophage expansion in female mice.

Notably, DRG sensory neuron-derived CCL2, a potent CCR2 ligand,^35^ was previously suggested to potentiate proliferation of resident macrophages and recruitment of blood monocytes to the injured DRG and peripheral nerve.^12,22^ We found that the macrophage expansion was not compromised in both male and female animals globally lacking CCL2. In addition, SNI-induced mechanical hypersensitivity was comparable in male and female mice lacking CCL2. It is unlikely that CCL2 regulates spinal microglia in which the CCL2 receptor, CCR2, is not expressed.^29^

IL34, the other cognate ligand for CSF1R, has recently reemerged as a potential candidate that instructs expansion of both microglia and macrophages. As discussed above, IL34 contributes to CNS microglia homeostasis.^14,73^ In the absence of IL-34, adult murine microglia number is significantly reduced. Furthermore, Wang et al.^72^ recently re-examined *Il34* knock-out mice and reported a more than 35% reduction in macrophage density in the uninjured DRG. Interestingly, Schwann cells were identified as the source of the IL-34, at least by in situ hybridization. Unfortunately, only male mice were examined. It remains to be determined whether the pain phenotype in both male and female mice is impacted equivalently by the loss of IL-34.

Taken together, our studies demonstrate that DRG sensory neuron-derived CSF1, by engaging the CSF1R that is expressed by both microglia and macrophages, triggers nerve injury-induced expansion of both resident microglia in the spinal cord and macrophages in the DRG. Based on these findings, we propose that a dual and likely contemporaneous process occurs in parallel in which both peripheral DRG macrophages and microglia contribute to neuropathic pain development. Notably, both cell populations have been implicated in pain chronicity.^52,80^ However, it seems that in female mice, DRG macrophages exert a more prominent role as first responders to nerve injury.

**Figure 1.**
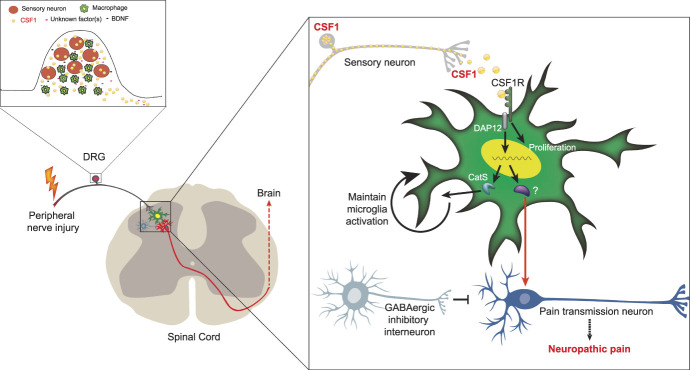
Sensory neuron-derived CSF1 triggers nerve injury-induced expansion of both resident microglia in the spinal cord and macrophages in the DRG. Peripheral nerve injury induces de novo expression of CSF1 in injured sensory neurons. The CSF1, in turn, is released from DRG neurons and stimulates proliferation of surrounding macrophages. The CSF1 is also transported to the spinal cord dorsal horn, where it engages the CSF1 receptor and stimulates microglia. The activated microglia undergo a DAP12-independent pathway that induces microglia proliferation and a DAP12-dependent pathway that induces expression of a host of neuropathic pain–associated genes. The figure is adapted from our previous article.^16^ DRG, dorsal root ganglion; CSF1, colony-stimulating factor 1.

In conclusion (Fig. 1), the CSF1 cytokine is de novo induced in DRG sensory neurons after peripheral nerve injury. The CSF1 is then transported along dorsal root axons to the dorsal horn, where it binds to microglial CSF1R and activates and upregulates a host of microglial genes. Engagement of the microglia, through a DAP12-dependent pathway, is a critical contributor to the consequent nerve injury-induced neuropathic pain behavior. In addition to activating the microglia, CSF1 induces microglial proliferation through a pathway that is independent of DAP12. Paralleling these events, CSF1 is also required for injury-induced macrophage expansion in the DRG, but this only occurs in male mice. Given the considerable evidence that interactions among neurons and immune cells are important contributors to the induction and maintenance of neuropathic pain after peripheral nerve injury,^27^ it is clear that a better understanding of the downstream biochemical pathways engaged by CSF1, in both microglia and macrophages, will provide new information concerning the mechanism through which nerve injury contributes to the transition from acute to persistent neuropathic pain, potentially providing novel treatment targets for chronic pain management.

## Disclosures

The authors have no conflicts of interest to declare.

A patent “Targeted Disruption of a CSF1-DAP12 Pathway Member Gene for the Treatment of Neuropathic Pain” has been filed by the authors.

## References

[1] Barclay J Clark AK Ganju P Gentry C Patel S Wotherspoon G Buxton F Song C Ullah J Winter J Fox A Bevan S Malcangio M. Role of the cysteine protease cathepsin S in neuropathic hyperalgesia. PAIN 2007;130:225–34. 17250968 10.1016/j.pain.2006.11.017

[2] Basbaum AI Bautista DM Scherrer G Julius D. Cellular and molecular mechanisms of pain. Cell 2009;139:267–84. 19837031 10.1016/j.cell.2009.09.028 PMC2852643

[3] Burnett SH Kershen EJ Zhang J Zeng L Straley SC Kaplan AM Cohen DA. Conditional macrophage ablation in transgenic mice expressing a Fas-based suicide gene. J Leukoc Biol 2004;75:612–23. 14726498 10.1189/jlb.0903442

[4] Chen G Zhang YQ Qadri YJ Serhan CN Ji RR. Microglia in pain: detrimental and protective roles in pathogenesis and resolution of pain. Neuron 2018;100:1292–311. 30571942 10.1016/j.neuron.2018.11.009 PMC6312407

[5] Chen O Donnelly CR Ji RR. Regulation of pain by neuro-immune interactions between macrophages and nociceptor sensory neurons. Curr Opin Neurobiol 2020;62:17–25. 31809997 10.1016/j.conb.2019.11.006 PMC7266706

[6] Cobos EJ Nickerson CA Gao F Chandran V Bravo-Caparros I Gonzalez-Cano R Riva P Andrews NA Latremoliere A Seehus CR Perazzoli G Nieto FR Joller N Painter MW Ma CHE Omura T Chesler EJ Geschwind DH Coppola G Rangachari M Woolf CJ Costigan M. Mechanistic differences in neuropathic pain modalities revealed by correlating behavior with global expression profiling. Cell Rep 2018;22:1301–12. 29386116 10.1016/j.celrep.2018.01.006 PMC5908229

[7] Colburn RW Rickman AJ DeLeo JA. The effect of site and type of nerve injury on spinal glial activation and neuropathic pain behavior. Exp Neurol 1999;157:289–304. 10364441 10.1006/exnr.1999.7065

[8] Duffield JS Forbes SJ Constandinou CM Clay S Partolina M Vuthoori S Wu S Lang R Iredale JP. Selective depletion of macrophages reveals distinct, opposing roles during liver injury and repair. J Clin Invest 2005;115:56–65. 15630444 10.1172/JCI22675 PMC539199

[9] Elmore MR Najafi AR Koike MA Dagher NN Spangenberg EE Rice RA Kitazawa M Matusow B Nguyen H West BL Green KN. Colony-stimulating factor 1 receptor signaling is necessary for microglia viability, unmasking a microglia progenitor cell in the adult brain. Neuron 2014;82:380–97. 24742461 10.1016/j.neuron.2014.02.040 PMC4161285

[10] Erblich B Zhu L Etgen AM Dobrenis K Pollard JW. Absence of colony stimulation factor-1 receptor results in loss of microglia, disrupted brain development and olfactory deficits. PLoS One 2011;6:e26317. 22046273 10.1371/journal.pone.0026317 PMC3203114

[11] Ginhoux F Greter M Leboeuf M Nandi S See P Gokhan S Mehler MF Conway SJ Ng LG Stanley ER Samokhvalov IM Merad M. Fate mapping analysis reveals that adult microglia derive from primitive macrophages. Science 2010;330:841–5. 20966214 10.1126/science.1194637 PMC3719181

[12] Ginhoux F Guilliams M. Tissue-resident macrophage ontogeny and homeostasis. Immunity 2016;44:439–49. 26982352 10.1016/j.immuni.2016.02.024

[13] Goren I Allmann N Yogev N Schurmann C Linke A Holdener M Waisman A Pfeilschifter J Frank S. A transgenic mouse model of inducible macrophage depletion: effects of diphtheria toxin-driven lysozyme M-specific cell lineage ablation on wound inflammatory, angiogenic, and contractive processes. Am J Pathol 2009;175:132–47. 19528348 10.2353/ajpath.2009.081002 PMC2708801

[14] Greter M Lelios I Pelczar P Hoeffel G Price J Leboeuf M Kundig TM Frei K Ginhoux F Merad M Becher B. Stroma-derived interleukin-34 controls the development and maintenance of langerhans cells and the maintenance of microglia. Immunity 2012;37:1050–60. 23177320 10.1016/j.immuni.2012.11.001 PMC4291117

[15] Gu N Peng J Murugan M Wang X Eyo UB Sun D Ren Y DiCicco-Bloom E Young W Dong H Wu LJ. Spinal microgliosis due to resident microglial proliferation is required for pain hypersensitivity after peripheral nerve injury. Cell Rep 2016;16:605–14. 27373153 10.1016/j.celrep.2016.06.018 PMC4956495

[16] Guan Z Kuhn JA Wang X Colquitt B Solorzano C Vaman S Guan AK Evans-Reinsch Z Braz J Devor M Abboud-Werner SL Lanier LL Lomvardas S Basbaum AI. Injured sensory neuron-derived CSF1 induces microglial proliferation and DAP12-dependent pain. Nat Neurosci 2016;19:94–101. 26642091 10.1038/nn.4189 PMC4703328

[17] Guimaraes RM Davoli-Ferreira M Fonseca MM Damasceno LEA Santa-Cecilia FV Kusuda R Menezes GB Cunha FQ Alves-Filho JC Cunha TM. Frontline Science: blood-circulating leukocytes fail to infiltrate the spinal cord parenchyma after spared nerve injury. J Leukoc Biol 2019;106:541–51. 31150565 10.1002/JLB.HI1118-458R

[18] Harris SE MacDougall M Horn D Woodruff K Zimmer SN Rebel VI Fajardo R Feng JQ Gluhak-Heinrich J Harris MA Abboud Werner S. Meox2Cre-mediated disruption of CSF-1 leads to osteopetrosis and osteocyte defects. Bone 2012;50:42–53. 21958845 10.1016/j.bone.2011.09.038 PMC3374485

[19] Heppner FL Greter M Marino D Falsig J Raivich G Hovelmeyer N Waisman A Rulicke T Prinz M Priller J Becher B Aguzzi A. Experimental autoimmune encephalomyelitis repressed by microglial paralysis. Nat Med 2005;11:146–52. 15665833 10.1038/nm1177

[20] Hickman SE Kingery ND Ohsumi TK Borowsky ML Wang LC Means TK El Khoury J. The microglial sensome revealed by direct RNA sequencing. Nat Neurosci 2013;16:1896–905. 24162652 10.1038/nn.3554 PMC3840123

[21] Hu LY Zhou Y Cui WQ Hu XM Du LX Mi WL Chu YX Wu GC Wang YQ Mao-Ying QL. Triggering receptor expressed on myeloid cells 2 (TREM2) dependent microglial activation promotes cisplatin-induced peripheral neuropathy in mice. Brain Behav Immun 2018;68:132–45. 29051087 10.1016/j.bbi.2017.10.011

[22] Hu P McLachlan EM. Distinct functional types of macrophage in dorsal root ganglia and spinal nerves proximal to sciatic and spinal nerve transections in the rat. Exp Neurol 2003;184:590–605. 14769352 10.1016/S0014-4886(03)00307-8

[23] Huang Y Xu Z Xiong S Sun F Qin G Hu G Wang J Zhao L Liang YX Wu T Lu Z Humayun MS So KF Pan Y Li N Yuan TF Rao Y Peng B. Repopulated microglia are solely derived from the proliferation of residual microglia after acute depletion. Nat Neurosci 2018;21:530–40. 29472620 10.1038/s41593-018-0090-8

[24] Hume DA MacDonald KP. Therapeutic applications of macrophage colony-stimulating factor-1 (CSF-1) and antagonists of CSF-1 receptor (CSF-1R) signaling. Blood 2012;119:1810–20. 22186992 10.1182/blood-2011-09-379214

[25] Inoue K Tsuda M. Microglia in neuropathic pain: cellular and molecular mechanisms and therapeutic potential. Nat Rev Neurosci 2018;19:138–52. 29416128 10.1038/nrn.2018.2

[26] Institute of Medicine (US) Committee on Advancing Pain Research C, and Education. Relieving pain in America: A blueprint for transforming prevention, care, education, and research. Washington: National Academies Press, 2011. 22553896

[27] Ji RR Chamessian A Zhang YQ. Pain regulation by non-neuronal cells and inflammation. Science 2016;354:572–7. 27811267 10.1126/science.aaf8924 PMC5488328

[28] Jin SX Zhuang ZY Woolf CJ Ji RR. p38 mitogen-activated protein kinase is activated after a spinal nerve ligation in spinal cord microglia and dorsal root ganglion neurons and contributes to the generation of neuropathic pain. J Neurosci 2003;23:4017–22. 12764087 10.1523/JNEUROSCI.23-10-04017.2003 PMC6741086

[29] Jung H Bhangoo S Banisadr G Freitag C Ren D White FA Miller RJ. Visualization of chemokine receptor activation in transgenic mice reveals peripheral activation of CCR2 receptors in states of neuropathic pain. J Neurosci 2009;29:8051–62. 19553445 10.1523/JNEUROSCI.0485-09.2009 PMC3097108

[30] Kalla R Liu Z Xu S Koppius A Imai Y Kloss CU Kohsaka S Gschwendtner A Moller JC Werner A Raivich G. Microglia and the early phase of immune surveillance in the axotomized facial motor nucleus: impaired microglial activation and lymphocyte recruitment but no effect on neuronal survival or axonal regeneration in macrophage-colony stimulating factor-deficient mice. J Comp Neurol 2001;436:182–201. 11438923

[31] Kobayashi M Konishi H Sayo A Takai T Kiyama H. TREM2/DAP12 signal elicits proinflammatory response in microglia and exacerbates neuropathic pain. J Neurosci 2016;36:11138–50. 27798193 10.1523/JNEUROSCI.1238-16.2016 PMC6705657

[32] Kobayashi M Konishi H Takai T Kiyama H. A DAP12-dependent signal promotes pro-inflammatory polarization in microglia following nerve injury and exacerbates degeneration of injured neurons. Glia 2015;63:1073–82. 25690660 10.1002/glia.22802

[33] Kondo Y Duncan ID. Selective reduction in microglia density and function in the white matter of colony-stimulating factor-1-deficient mice. J Neurosci Res 2009;87:2686–95. 19396881 10.1002/jnr.22096 PMC4843845

[34] Korczeniewska OA Husain S Khan J Eliav E Soteropoulos P Benoliel R. Differential gene expression in trigeminal ganglia of male and female rats following chronic constriction of the infraorbital nerve. Eur J Pain 2018;22:875–88. 29350446 10.1002/ejp.1174

[35] Kwon MJ Shin HY Cui Y Kim H Thi AH Choi JY Kim EY Hwang DH Kim BG. CCL2 mediates neuron-macrophage interactions to drive proregenerative macrophage activation following preconditioning injury. J Neurosci 2015;35:15934–47. 26631474 10.1523/JNEUROSCI.1924-15.2015 PMC6605453

[36] Lanier LL. DAP10- and DAP12-associated receptors in innate immunity. Immunol Rev 2009;227:150–60. 19120482 10.1111/j.1600-065X.2008.00720.x PMC2794881

[37] Lee S Shi XQ Fan A West B Zhang J. Targeting macrophage and microglia activation with colony stimulating factor 1 receptor inhibitor is an effective strategy to treat injury-triggered neuropathic pain. Mol Pain 2018;14:1744806918764979. 29546785 10.1177/1744806918764979 PMC5858622

[38] Lim H Lee H Noh K Lee SJ. IKK/NF-kappaB-dependent satellite glia activation induces spinal cord microglia activation and neuropathic pain after nerve injury. PAIN 2017;158:1666–77. 28722693 10.1097/j.pain.0000000000000959

[39] Lin H Lee E Hestir K Leo C Huang M Bosch E Halenbeck R Wu G Zhou A Behrens D Hollenbaugh D Linnemann T Qin M Wong J Chu K Doberstein SK Williams LT. Discovery of a cytokine and its receptor by functional screening of the extracellular proteome. Science 2008;320:807–11. 18467591 10.1126/science.1154370

[40] Liu L Persson JK Svensson M Aldskogius H. Glial cell responses, complement, and clusterin in the central nervous system following dorsal root transection. Glia 1998;23:221–38. 9633807

[41] Malcangio M. Role of the immune system in neuropathic pain. Scand J Pain 2019;20:33–7. 31730538 10.1515/sjpain-2019-0138

[42] McVicar DW Trinchieri G. CSF-1R, DAP12 and beta-catenin: a menage a trois. Nat Immunol 2009;10:681–3. 19536190 10.1038/ni0709-681

[43] Metcalf D. The colony-stimulating factors and cancer. Nat Rev Cancer 2010;10:425–34. 20495576 10.1038/nrc2843 PMC3345291

[44] Nandi S Cioce M Yeung YG Nieves E Tesfa L Lin H Hsu AW Halenbeck R Cheng HY Gokhan S Mehler MF Stanley ER. Receptor-type protein-tyrosine phosphatase zeta is a functional receptor for interleukin-34. J Biol Chem 2013;288:21972–86. 23744080 10.1074/jbc.M112.442731 PMC3724651

[45] Nandi S Gokhan S Dai XM Wei S Enikolopov G Lin H Mehler MF Stanley ER. The CSF-1 receptor ligands IL-34 and CSF-1 exhibit distinct developmental brain expression patterns and regulate neural progenitor cell maintenance and maturation. Dev Biol 2012;367:100–13. 22542597 10.1016/j.ydbio.2012.03.026 PMC3388946

[46] Nguyen MQ Le Pichon CE Ryba N. Stereotyped transcriptomic transformation of somatosensory neurons in response to injury. Elife 2019;8:e49679. 31592768 10.7554/eLife.49679 PMC6783272

[47] Noh MC Mikler B Joy T Smith PA. Time course of inflammation in dorsal root ganglia correlates with differential reversibility of mechanical allodynia. Neuroscience 2020;428:199–216. 31918012 10.1016/j.neuroscience.2019.12.040

[48] Okubo M Yamanaka H Kobayashi K Dai Y Kanda H Yagi H Noguchi K. Macrophage-colony stimulating factor derived from injured primary afferent induces proliferation of spinal microglia and neuropathic pain in rats. PLoS One 2016;11:e0153375. 27071004 10.1371/journal.pone.0153375 PMC4829214

[49] Otero K Turnbull IR Poliani PL Vermi W Cerutti E Aoshi T Tassi I Takai T Stanley SL Miller M Shaw AS Colonna M. Macrophage colony-stimulating factor induces the proliferation and survival of macrophages via a pathway involving DAP12 and beta-catenin. Nat Immunol 2009;10:734–43. 19503107 10.1038/ni.1744 PMC4004764

[50] Parkhurst CN Yang G Ninan I Savas JN Yates JR III Lafaille JJ Hempstead BL Littman DR Gan WB. Microglia promote learning-dependent synapse formation through brain-derived neurotrophic factor. Cell 2013;155:1596–609. 24360280 10.1016/j.cell.2013.11.030 PMC4033691

[51] Peng J Gu N Zhou L B Eyo U Murugan M Gan WB Wu LJ. Microglia and monocytes synergistically promote the transition from acute to chronic pain after nerve injury. Nat Commun 2016;7:12029. 27349690 10.1038/ncomms12029 PMC4931235

[52] Price TJ Basbaum AI Bresnahan J Chambers JF De Koninck Y Edwards RR Ji RR Katz J Kavelaars A Levine JD Porter L Schechter N Sluka KA Terman GW Wager TD Yaksh TL Dworkin RH. Transition to chronic pain: opportunities for novel therapeutics. Nat Rev Neurosci 2018;19:383–4. 29765159 10.1038/s41583-018-0012-5 PMC6237656

[53] Pyonteck SM Akkari L Schuhmacher AJ Bowman RL Sevenich L Quail DF Olson OC Quick ML Huse JT Teijeiro V Setty M Leslie CS Oei Y Pedraza A Zhang J Brennan CW Sutton JC Holland EC Daniel D Joyce JA. CSF-1R inhibition alters macrophage polarization and blocks glioma progression. Nat Med 2013;19:1264–72. 24056773 10.1038/nm.3337 PMC3840724

[54] Raghavendra V Tanga F DeLeo JA. Inhibition of microglial activation attenuates the development but not existing hypersensitivity in a rat model of neuropathy. J Pharmacol Exp Ther 2003;306:624–30. 12734393 10.1124/jpet.103.052407

[55] Raivich G Haas S Werner A Klein MA Kloss C Kreutzberg GW. Regulation of MCSF receptors on microglia in the normal and injured mouse central nervous system: a quantitative immunofluorescence study using confocal laser microscopy. J Comp Neurol 1998;395:342–58. 9596528 10.1002/(sici)1096-9861(19980808)395:3<342::aid-cne6>3.0.co;2-2

[56] Reinhold AK Rittner HL. Characteristics of the nerve barrier and the blood dorsal root ganglion barrier in health and disease. Exp Neurol 2020;327:113244. 32057794 10.1016/j.expneurol.2020.113244

[57] Renthal W Tochitsky I Yang L Cheng YC Li E Kawaguchi R Geschwind DH Woolf CJ. Transcriptional reprogramming of distinct peripheral sensory neuron subtypes after axonal injury. Neuron 2020;108:128–44.e129. 32810432 10.1016/j.neuron.2020.07.026 PMC7590250

[58] Rutkowski MD Pahl JL Sweitzer S van Rooijen N DeLeo JA. Limited role of macrophages in generation of nerve injury-induced mechanical allodynia. Physiol Behav 2000;71:225–35. 11150554 10.1016/s0031-9384(00)00333-4

[59] Saederup N Cardona AE Croft K Mizutani M Cotleur AC Tsou CL Ransohoff RM Charo IF. Selective chemokine receptor usage by central nervous system myeloid cells in CCR2-red fluorescent protein knock-in mice. PLoS One 2010;5:e13693. 21060874 10.1371/journal.pone.0013693 PMC2965160

[60] Sawada M Suzumura A Yamamoto H Marunouchi T. Activation and proliferation of the isolated microglia by colony stimulating factor-1 and possible involvement of protein kinase C. Brain Res 1990;509:119–24. 2306629 10.1016/0006-8993(90)90317-5

[61] Schreiber RC Shadiack AM Bennett TA Sedwick CE Zigmond RE. Changes in the macrophage population of the rat superior cervical ganglion after postganglionic nerve injury. J Neurobiol 1995;27:141–53. 7658197 10.1002/neu.480270203

[62] Schulz C Gomez Perdiguero E Chorro L Szabo-Rogers H Cagnard N Kierdorf K Prinz M Wu B Jacobsen SE Pollard JW Frampton J Liu KJ Geissmann F. A lineage of myeloid cells independent of Myb and hematopoietic stem cells. Science 2012;336:86–90. 22442384 10.1126/science.1219179

[63] Shepherd AJ Mickle AD Golden JP Mack MR Halabi CM de Kloet AD Samineni VK Kim BS Krause EG RWt Gereau Mohapatra DP. Macrophage angiotensin II type 2 receptor triggers neuropathic pain. Proc Natl Acad Sci U S A 2018;115:E8057–66. 30082378 10.1073/pnas.1721815115 PMC6112686

[64] Sieweke MH Allen JE. Beyond stem cells: self-renewal of differentiated macrophages. Science 2013;342:1242974. 24264994 10.1126/science.1242974

[65] Simeoli R Montague K Jones HR Castaldi L Chambers D Kelleher JH Vacca V Pitcher T Grist J Al-Ahdal H Wong LF Perretti M Lai J Mouritzen P Heppenstall P Malcangio M. Exosomal cargo including microRNA regulates sensory neuron to macrophage communication after nerve trauma. Nat Commun 2017;8:1778. 29176651 10.1038/s41467-017-01841-5 PMC5701122

[66] Sorge RE Mapplebeck JC Rosen S Beggs S Taves S Alexander JK Martin LJ Austin JS Sotocinal SG Chen D Yang M Shi XQ Huang H Pillon NJ Bilan PJ Tu Y Klip A Ji RR Zhang J Salter MW Mogil JS. Different immune cells mediate mechanical pain hypersensitivity in male and female mice. Nat Neurosci 2015;18:1081–3. 26120961 10.1038/nn.4053 PMC4772157

[67] Spangenberg E Severson PL Hohsfield LA Crapser J Zhang J Burton EA Zhang Y Spevak W Lin J Phan NY Habets G Rymar A Tsang G Walters J Nespi M Singh P Broome S Ibrahim P Zhang C Bollag G West BL Green KN. Sustained microglial depletion with CSF1R inhibitor impairs parenchymal plaque development in an Alzheimer's disease model. Nat Commun 2019;10:3758. 31434879 10.1038/s41467-019-11674-z PMC6704256

[68] Stanley ER Cifone M Heard PM Defendi V. Factors regulating macrophage production and growth: identity of colony-stimulating factor and macrophage growth factor. J Exp Med 1976;143:631–47. 1082493 10.1084/jem.143.3.631 PMC2190132

[69] Stephens KE Zhou W Ji Z Chen Z He S Ji H Guan Y Taverna SD. Sex differences in gene regulation in the dorsal root ganglion after nerve injury. BMC Genomics 2019;20:147. 30782122 10.1186/s12864-019-5512-9 PMC6381758

[70] Tashima R Mikuriya S Tomiyama D Shiratori-Hayashi M Yamashita T Kohro Y Tozaki-Saitoh H Inoue K Tsuda M. Bone marrow-derived cells in the population of spinal microglia after peripheral nerve injury. Sci Rep 2016;6:23701. 27005516 10.1038/srep23701 PMC4804310

[71] Ulland TK Colonna M. TREM2—a key player in microglial biology and Alzheimer disease. Nat Rev Neurol 2018;14:667–75. 30266932 10.1038/s41582-018-0072-1

[72] Wang PL Yim AKY Kim KW Avey D Czepielewski RS Colonna M Milbrandt J Randolph GJ. Peripheral nerve resident macrophages share tissue-specific programming and features of activated microglia. Nat Commun 2020;11:2552. 32439942 10.1038/s41467-020-16355-w PMC7242366

[73] Wang Y Szretter KJ Vermi W Gilfillan S Rossini C Cella M Barrow AD Diamond MS Colonna M. IL-34 is a tissue-restricted ligand of CSF1R required for the development of Langerhans cells and microglia. Nat Immunol 2012;13:753–60. 22729249 10.1038/ni.2360 PMC3941469

[74] Wei S Nandi S Chitu V Yeung YG Yu W Huang M Williams LT Lin H Stanley ER. Functional overlap but differential expression of CSF-1 and IL-34 in their CSF-1 receptor-mediated regulation of myeloid cells. J Leukoc Biol 2010;88:495–505. 20504948 10.1189/jlb.1209822 PMC2924605

[75] Wlaschin JJ Gluski JM Nguyen E Silberberg H Thompson JH Chesler AT Le Pichon CE. Dual leucine zipper kinase is required for mechanical allodynia and microgliosis after nerve injury. Elife 2018;7. 10.7554/eLife.33910 PMC6029846 29968565

[76] Wright DE Wagers AJ Gulati AP Johnson FL Weissman IL. Physiological migration of hematopoietic stem and progenitor cells. Science 2001;294:1933–6. 11729320 10.1126/science.1064081

[77] Yang G Chen L Gao Z Wang Y. Implication of microglia activation and CSF-1/CSF-1Rpathway in lumbar disc degeneration-related back pain. Mol Pain 2018;14:1744806918811238. 30326776 10.1177/1744806918811238 PMC6243401

[78] Yang G Tan Q Li Z Liu K Wu J Ye W Mei H Yu H. The AMPK pathway triggers autophagy during CSF1-induced microglial activation and may be implicated in inducing neuropathic pain. J Neuroimmunol 2020;345:577261. 32570135 10.1016/j.jneuroim.2020.577261

[79] Yoshida H Hayashi S Kunisada T Ogawa M Nishikawa S Okamura H Sudo T Shultz LD Nishikawa S. The murine mutation osteopetrosis is in the coding region of the macrophage colony stimulating factor gene. Nature 1990;345:442–4. 2188141 10.1038/345442a0

[80] Yu X Liu H Hamel KA Morvan MG Yu S Leff J Guan Z Braz JM Basbaum AI. Dorsal root ganglion macrophages contribute to both the initiation and persistence of neuropathic pain. Nat Commun 2020;11:264. 31937758 10.1038/s41467-019-13839-2 PMC6959328

[81] Zhan L Krabbe G Du F Jones I Reichert MC Telpoukhovskaia M Kodama L Wang C Cho SH Sayed F Li Y Le D Zhou Y Shen Y West B Gan L. Proximal recolonization by self-renewing microglia re-establishes microglial homeostasis in the adult mouse brain. PLoS Biol 2019;17:e3000134. 30735499 10.1371/journal.pbio.3000134 PMC6383943

[82] Zhou LJ Peng J Xu YN Zeng WJ Zhang J Wei X Mai CL Lin ZJ Liu Y Murugan M Eyo UB Umpierre AD Xin WJ Chen T Li M Wang H Richardson JR Tan Z Liu XG Wu LJ. Microglia are indispensable for synaptic plasticity in the spinal dorsal horn and chronic pain. Cell Rep 2019;27:3844–59 e3846. 31242418 10.1016/j.celrep.2019.05.087 PMC7060767

[83] Zigmond RE Echevarria FD. Macrophage biology in the peripheral nervous system after injury. Prog Neurobiol 2019;173:102–21. 30579784 10.1016/j.pneurobio.2018.12.001 PMC6340791

[84] Zurborg S Piszczek A Martinez C Hublitz P Al Banchaabouchi M Moreira P Perlas E Heppenstall PA. Generation and characterization of an Advillin-Cre driver mouse line. Mol Pain 2011;7:66. 21906401 10.1186/1744-8069-7-66 PMC3185264

